# Impact of enteral feeding strategies on nosocomial *Clostridioides difficile* infection-induced diarrhea

**DOI:** 10.3389/fnut.2026.1781944

**Published:** 2026-03-17

**Authors:** Ghada Alasbly, Sara Alotaishan, Yasmin Algindan, Rabie Khattab

**Affiliations:** Department of Clinical Nutrition, Imam Abdulrahman Bin Faisal University, Dammam, Saudi Arabia

**Keywords:** biochemical markers, closed system, *Clostridioides difficile* infection, continuous feeding, enteral feeding, intermittent feeding, open system, Saudi Arabia

## Abstract

**Background:**

*Clostridioides difficile* infection (CDI) is a leading cause of healthcare-associated diarrhea, associated with prolonged hospitalization, increased costs, and higher mortality. Diarrhea severity is influenced by host factors, biochemical markers, and nutritional support. Although enteral feeding is essential when oral intake is not feasible, the impact of feeding mode and system type on CDI-related diarrhea remains underexplored. This study examined associations between enteral feeding strategies and diarrhea severity, along with relevant biochemical markers and medication use, particularly antibiotics and proton pump inhibitors.

**Methods:**

A retrospective cross-sectional investigation was conducted on adult CDI patients across tertiary hospitals in Saudi Arabia’s Eastern Province (Jan 2020–May 2025). Of 97 records, 78 met inclusion criteria. Demographic, nutritional, and biochemical data—including sodium, creatinine, albumin, and hemoglobin—were analyzed.

**Results:**

All cases ranged from mild to moderate diarrhea; males comprised 57.1%. Continuous feeding strongly associated with milder diarrhea compared to intermittent feeding (OR = 7.91; 95% CI: 2.688–23.253; *p* < 0.001). Feeding system type showed a non-significant trend, with open systems linked to more moderate cases. Continuous feeding stabilized sodium, while intermittent feeding correlated with elevated creatinine and reduced albumin/hemoglobin. Leukocytosis, hypoalbuminemia, and creatinine elevation emerged as prognostic markers. Over 90% of patients received antibiotics and PPIs concurrently, underscoring stewardship needs.

**Conclusion:**

Continuous enteral feeding appears clinically advantageous in CDI, improving tolerance and biochemical stability. Findings highlight the importance of nutritional monitoring, electrolyte management, and rational prescribing. Region-specific evidence contributes to global CDI literature and supports future prospective studies to refine feeding strategies.

## 1 Introduction

*Clostridioides difficile* infection (CDI) remains a significant healthcare-associated concern, particularly among patients receiving enteral feeding (EF). CDI leads to toxin-mediated colonic inflammation and diarrhea, contributing substantially to morbidity and mortality in hospitalized patients (1). Individuals on EF are at increased risk, with diarrhea reported in up to 60% of cases (2), largely due to formula or equipment contamination and EF-related alterations in gut microbiota. CDI incidence among enterally fed patients ranges from 8 to 20%, with EF nearly doubling infection risk (3, 4). Disruption of the gut microbiome and mucosal immunity facilitates *C. difficile* colonization (5). Clinical severity varies from mild diarrhea to severe complications, including dehydration, electrolyte imbalance, and toxic megacolon (6).

Enteral nutrition is administered when oral intake is inadequate, commonly via continuous feeding (CF) or intermittent feeding (IF) strategies (7). Key CDI risk factors include antibiotic exposure, proton pump inhibitor (PPI) use, poor hygiene practices, and EF (8). However, direct comparisons of EF strategies and their impact on CDI-related diarrhea remain limited.

Patients receiving EF have nearly twice the CDI risk of non-fed patients, with approximately 11% of ICU-related diarrhea attributable to CDI (4, 9, 10). This risk is linked to altered gut motility, pH, and microbial composition. Fiber-enriched formulas reduce CDI incidence and symptom severity compared with fiber-free feeds (5, 11). Additional risk factors include advanced age, chronic illness, antibiotic use, and immunosuppression (12–14).

EF-related infections arise from microbiota imbalance, reduced motility, and biofilm formation on feeding tubes, compounded by poor handling, prolonged hang times, critical illness, and gut hypoperfusion (5, 15, 16). CDI-associated diarrhea is often accompanied by biochemical disturbances, including hypoalbuminemia, leukocytosis, glucose variability, electrolyte imbalance, and elevated blood urea nitrogen (BUN) and creatinine, reflecting dehydration or renal impairment (17–23).

CDI risk is further influenced by formula composition, feeding hygiene, patient characteristics, and medication exposure. Antibiotics—particularly cephalosporins and fluoroquinolones—and acid-suppressive agents remain the strongest contributors (5, 24–26). Preventive strategies include antimicrobial stewardship, reduced PPI use, strict infection control, aseptic feeding practices, fiber-enriched formulas, and probiotics, which may reduce CDI incidence by up to 50% (4, 27–33).

The primary objective of this study is to evaluate the impact of different EF strategies on the severity of nosocomial CDI-induced diarrhea. The study further investigates associated changes in clinical and biochemical indices, including albumin, blood glucose, complete blood count, electrolytes, and hydration markers such as blood urea nitrogen and creatinine.

## 2 Methods and procedures

### 2.1 Study design

This is an observational, cross-sectional, retrospective study that assesses the impact of EF strategies on nosocomial CDI-related diarrhea through the collection and analysis of existing patient medical records from hospitals across the Eastern Region of Saudi Arabia. The research was conducted in accordance with the ethical standards and regulations established by the Institutional Review Boards (IRBs) of the university and the participating hospitals.

### 2.2 Sample size calculation

A total sample size of 64 was calculated using the formula 𝑛 = $\frac{Z^{2}*p\;\left(1-p\right)}{d^{2}}$ , where n represents the sample size, *Z* (1.96) is the statistic corresponding to a 95% level of confidence, *p* (9.1%) is the expected average prevalence of CDI derived from different studies (3, 5, 34), and *d* (7%) is the desired absolute precision.

### 2.3 Data collection method

The study involved a considerable number of hospitalized patients in four hospitals including King Fahd Hospital of the University (KFHU) in Khobar, King Fahd Military Medical Complex (KFMMC) in Dhahran, Dr. Sulaiman Al Habib Hospital (HMG) in Khobar, and King Fahad Specialist Hospital (KFSH) in Dammam. Using the hospitals’ advanced electronic medical record (EMR) systems, access to relevant data for the study was achieved seamlessly. A detailed search within the EMR systems was carried out to identify all patients who had CDI-induced diarrhea during their hospital stay, after which EF exposure was assessed. Their medical records were thoroughly reviewed to collect data on the key variables of interest.

### 2.4 Inclusion and exclusion criteria

Patients aged 18 years or older who had been hospitalized for at least 48 h before CDI diagnosis were included, ensuring the infection met criteria for a healthcare-associated case. Eligible patients were those who developed CDI-induced diarrhea during their hospital stay and received enteral feeding; all other patients were excluded.

### 2.5 Dependent and independent variables

The dependent variables included nutritional status indicators (e.g., serum albumin), feeding-related complications such as aspiration or intolerance, relevant laboratory parameters, length of hospital stay, and mortality. Independent variables comprised enteral feeding method (intermittent vs. continuous; open vs. closed systems), duration of enteral feeding, patient demographics (age and sex), and clinical background, including treatment and surgical history and prior use of antibiotics and PPIs.

### 2.6 Reliability and validity

The reliability of data collection was ensured through the training of the data collectors involved in the study and the standardization of the measurement tools they used. The authenticity of the data was confirmed by employing established evaluation instruments and adhering to standardized procedures.

### 2.7 Statistical analysis

The data analysis was conducted using the Statistical Package for the Social Sciences (SPSS) and Statistical Analysis System (SAS) to ensure a comprehensive evaluation. A significance level of *α* = 0.05 was adopted, with *p*-values less than 0.05 considered statistically significant. Confidence intervals and effect sizes were also assessed to provide a more complete interpretation of the findings.

Analyses examined associations between feeding methods (intermittent vs. continuous; open vs. closed systems) among hospitalized EF patients. Descriptive statistics summarized baseline characteristics. For outcome comparisons between groups, independent *t*-tests or Mann–Whitney U tests were used for continuous variables, and chi-square tests for categorical variables. Trends in nutritional and clinical parameters were illustrated using graphical methods, enabling evaluation of the impact of feeding strategies on patient outcomes.

### 2.8 Confidentiality, data security, and ethical considerations

Patient data were treated with strict confidentiality, accessible only to members of the research team directly involved in the study. All patient information was securely stored and used exclusively for the purposes of this research project. Data will be permanently deleted upon completion of the study. Ethical approvals were obtained with a focus on safeguarding patient privacy and confidentiality. Approvals were secured from King Fahd Hospital of the University through Imam Abdulrahman bin Faisal University Review Board (IRB-PGS-2024-03-546; July 21, 2024), the Armed Forces Hospitals Eastern Province Institutional Review Board (Protocol Number: AFHER-IRB-2025-02; December 23, 2024), Dr. Sulaiman Al Habib Medical Group (Study Number: RC24.12.96; January 31, 2025), and King Fahad Specialist Hospital (Study Number: EXT0448; December 31, 2024). No data were disclosed to third parties without consent.

## 3 Results

### 3.1 Selection process

**Figure 1:**
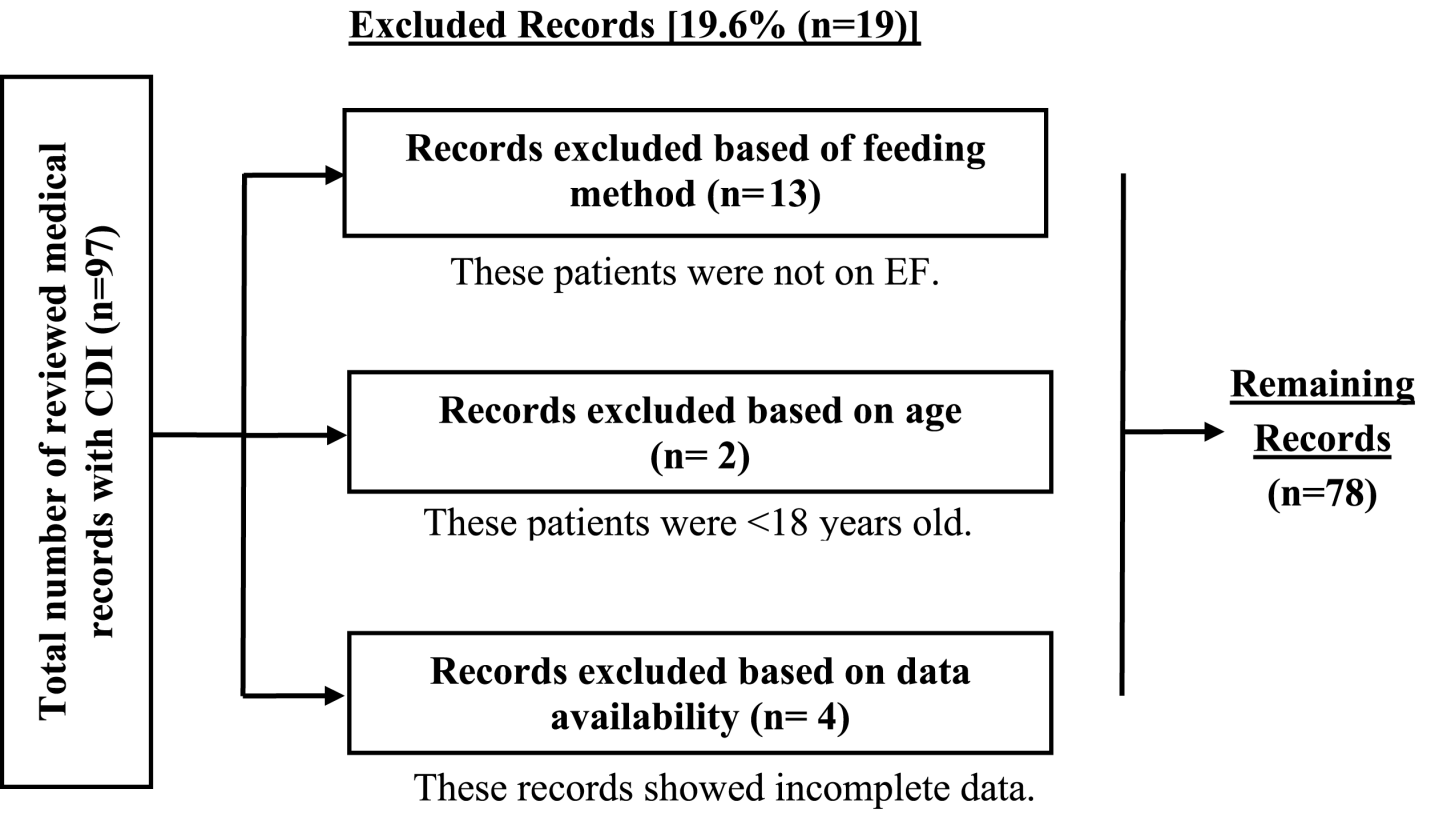
Screening of patients for potential inclusion in the study.

A total of 97 medical records of patients diagnosed with CDI-induced diarrhea were reviewed. After applying the inclusion criteria, 78 patients qualified for analysis (Figure 1). These patients had developed CDI-induced diarrhea during hospitalization, had been admitted for at least 48 h before diagnosis, and had received EF. The remaining 19 cases were excluded because they did not receive EF, were younger than 18 years, or had incomplete medical records.

### 3.2 Demographic and clinical characteristics of patients with CDI-induced diarrhea

The study included 78 patients with CDI-induced diarrhea, with a mean age of 66.3 ± 15.9 years. Of these, 57.1% (*n* = 44) were male and 42.9% (*n* = 33) were female. Regarding nutritional status, 39.7% of patients had a normal body mass index (BMI), while 32.9% were classified as overweight. Continuous feeding was applied in 53% of cases, whereas 47% received intermittent feeding (Table 1). The mean duration of enteral feeding was 1.6 ± 2.9 months.

**Table 1:** Descriptive statistics of collected variables.

	Frequency	Percentage
Gender		
Male	44	57.1
Female	33	42.9
BMI		
Underweight	4	5.5
Normal	29	39.7
Overweight	24	32.9
Obese	16	21.9
Feeding strategy		
Continuous	41	53
Intermittent	37	47
Feeding type		
Open	64	82.1
closed	14	17.9
Diarrhea severity		
Mild	29	37.2
Moderate	49	62.8
Severe	0	0
Aspiration		
Yes	2	2.6
No	76	97.4
Nutritional tolerance		
Yes	1	1.3
No	77	98.7
Antibiotics used		
Yes	70	89.7
No	8	10.3
PPI		
Yes	65	83.3
No	13	16.7

The severity of diarrhea was either mild (*n* = 29) or moderate (*n* = 49), with no cases classified as severe. The mean length of hospital stay was 38.6 ± 51.5 days. Additionally, antibiotic therapy was administered to 70 patients, while proton pump inhibitors (PPIs) were used in 65 patients (Table 1).

### 3.3 Impact of CDI on laboratory parameters among patients on different feeding strategies

Table 2 summarizes the changes in blood parameters among patients receiving a continuous feeding strategy before and after CDI, highlighting several significant alterations. Sodium levels increased significantly (139.9 ± 7.6 vs. 142.0 ± 7.2 mmol/L, *p* = 0.003). Chloride and potassium values remained within similar ranges but still showed statistically significant differences (*p* = 0.000 and *p* = 0.006, respectively), likely reflecting post-CDI variability. Albumin (27.4 ± 6.3 vs. 26.7 ± 9.0 g/L, *p* = 0.006) and BUN (15.3 ± 10.4 vs. 13.7 ± 11.1 mg/dL, *p* = 0.002) both decreased significantly, whereas creatinine increased markedly (117.2 ± 109.3 vs. 131.8 ± 113.2 μmol/L, *p* = 0.000), suggesting possible renal stress.

**Table 2:** Blood parameters of patients on continuous feeding/intermittent feeding.

Blood parameters	Continuous feeding			Intermittent feeding		
	Mean (SD)		*P*-value	Mean (SD)		*P*-value
	Before CDI	After CDI		Before CDI	After CDI	
Sodium	139.9 (7.6)	142.0 (7.2)	0.003	137.8 (7.3)	137.0 (6.2)	0.030
Potassium	3.9 (0.8)	3.9 (1.1)	0.006	3.9 (0.6)	3.9 (0.8)	0.367
Phosphate	1.1 (0.6)	1.4 (1.1)	0.157	1.2 (0.5)	1.1 (0.6)	0.698
Chloride	108.6 (9.7)	108.6 (8.3)	0.000	103.8 (9.2)	101.8 (9.4)	0.429
Albumin	27.4 (6.3)	26.7 (9.0)	0.006	29.8 (6.2)	28.9 (7.6)	0.001
Blood glucose	8.9 (4.0)	8.8 (4.1)	0.086	10.1 (4.1)	8.9 (2.4)	0.353
HGB	9.6 (1.8)	9.4 (2.3)	0.555	10.6 (2.2)	9.9 (1.8)	0.005
WBC	11.1 (8.9)	12.1 (7.6)	0.000	10.5 (7.7)	9.0 (4.0)	0.649
BUN	15.3 (10.4)	13.7 (11.1)	0.002	15.3 (17.9)	14.1 (17.1)	0.020
Creatinine	117.2 (109.3)	131.8 (113.2)	0.000	116.5 (121.8)	140.8 (148.2)	0.000

HGB, Hemoglobin; WBC, White blood cell; BUN, Blood urea nitrogen.

White blood cell (WBC) counts rose significantly (11.1 ± 8.9 vs. 12.1 ± 7.6 × 10^9^/L, *p* = 0.000), consistent with an infection-driven inflammatory response. Hemoglobin declined slightly but not significantly (*p* = 0.555), while phosphate levels rose slightly without statistical significance. Blood glucose remained stable (*p* = 0.086).

In summary, CDI in continuously fed patients was associated with significant increases in sodium, creatinine, and WBC counts, along with decreases in albumin and BUN, while other parameters showed no meaningful changes (Table 2).

Similarly, Table 2 summarizes the changes in blood parameters among patients receiving the intermittent feeding strategy. The results show that serum creatinine increased significantly after CDI, rising from 116.5 ± 121.8 to 140.8 ± 148.2 μmol/L (*p* = 0.000). In contrast, most other parameters demonstrated a decline in mean values following CDI. Significant reductions were observed in serum sodium (137.8 ± 7.3 to 137.0 ± 6.2 mmol/L, *p* = 0.030), albumin (29.8 ± 6.2 to 28.9 ± 7.6 g/L, *p* = 0.001), hemoglobin (10.6 ± 2.2 to 9.9 ± 1.8 g/dL, *p* = 0.005), and BUN (15.3 ± 17.9 to 14.1 ± 17.1 mg/dL, *p* = 0.020). Changes in potassium, chloride, phosphate, blood glucose, and WBC count were not statistically significant (*p* > 0.05), suggesting greater variability in these measures among intermittently fed patients. While the clinical implications of these findings require further exploration, the results highlight significant alterations in renal, nutritional, and hematological parameters associated with CDI in this group (Table 2).

The BUN/creatinine ratio was also assessed in relation to feeding strategy (Table 3). The mean ratio was 28.0 ± 12.4 among patients receiving intermittent feeding compared with 23.6 ± 13.1 in those on continuous feeding; however, this difference was not statistically significant. For further analysis, the BUN/creatinine ratio was categorized into three levels: <10, 10–20, and >20. Among patients on continuous feeding, 51.9% (*n* = 14) had a ratio greater than 20, whereas this proportion was higher in the intermittent feeding group, with 70.6% (*n* = 24) exceeding a ratio of 20 (Table 3).

**Table 3:** BUN/creatinine ratio in relation to feeding strategy.

Feeding strategy	Mean (SD)	*p*-value	<10 (n, %)	10–20 (n, %)	>20 (n, %)
Continuous	23.6 (13.1)	0.178	5 (18.5%)	8 (29.6%)	14 (51.9%)
Intermittent	28.0 (12.4)		3 (8.8%)	7 (20.6%)	24 (70.6%)

BUN, Blood urea nitrogen.

The relationship between antibiotic use and PPI therapy was also examined. A highly significant association was observed between the two variables, as 91.4% of patients receiving antibiotics were also prescribed PPI medication, while only 8.6% received antibiotics without PPIs (*p* = 0.000) (Table 4).

**Table 4:** Relationship between antibiotics and PPI medications.

Antibiotics used	PPI		*P*-value	Odds ratio	95% CI	
	Yes	No			Lower	Upper
Yes	64 (91.4%)	6 (8.6%)	0.000	74.7	7.82	712.83
No	1 (12.5%)	7 (87.5%)				
Total	65	13				

PPIs, Proton pump inhibitors; CI, Confidence interval.

### 3.4 Association between enteral feeding formula type and severity of diarrhea

The severity of diarrhea was examined in relation to the type of enteral formula used. Among patients receiving Ensure (± Plus), 50.0% experienced moderate diarrhea and 50.0% had mild diarrhea. In the Nepro HP group, 69.2% presented with moderate diarrhea compared to 30.8% with mild diarrhea. For those receiving Glucerna, 83.3% developed moderate diarrhea. Patients on Impact formulas (Enteral/AR) showed 44.4% with moderate diarrhea, while the Peptamen group (±AF/±Intense) had 57.1% with moderate diarrhea. Supportan users demonstrated 80.0% moderate diarrhea, Surenutri (±DM) users 100%, and Fresubin (Diben/Hepatic/Intensive) users 60%. Other formulas (Diason HP, Fibersource, Jevity, Pulmocare, etc.) were associated with 66.7% moderate diarrhea. Despite these variations, the difference in diarrhea severity across formula groups was not statistically significant (*p* = 0.617) (Table 5).

**Table 5:** Relationship between type of enteral formula and diarrhea severity in hospitalized patients.

Formula used categorical	Severity of diarrhea		*P*-value
	Mild	Moderate	
Ensure (±Plus)	7	7	0.617
Nepro HP	4	9	
Glucerna	1	5	
Impact (Enteral/AR)	5	4	
Peptamen (±AF/±Intense)	6	8	
Supportan	1	4	
Surenutri (± DM)	0	3	
Fresubin (Diben/Hepatic/Intensive)	2	3	
Others (Diason HP, Fibersource, Jevity, Pulmocare, etc.)	3	6	
Total	29	49	

### 3.5 Association between feeding strategy, feeding system, and diarrhea severity

This study evaluated the impact of both feeding strategy (continuous vs. intermittent) and feeding system (open vs. closed) on diarrhea severity in patients with CDI-induced diarrhea. The analysis revealed a highly significant association between feeding strategy and diarrhea severity (χ^2^ = 16.879; *p* < 0.001), which was further supported by Fisher’s exact and Likelihood Ratio tests (*p* = 0.000) (Table 6). Patients receiving continuous feeding were nearly eight times more likely to experience mild diarrhea compared to those on intermittent feeding (OR = 7.91; 95% CI: 2.688–23.253), with correlation coefficients indicating a moderate positive relationship (*r* = *ρ* = 0.451; *p* < 0.001).

**Table 6:** Association between feeding strategy/type and diarrhea severity.

Variable	Category	Mild diarrhea (*n* = 29)	Moderate diarrhea (*n* = 49)	*P*-value	Odds ratio	95% CI	
						Lower	Upper
Feeding strategy	Continuous	24 (83%)	17 (35%)	0.000	7.91	2.688	23.253
	Intermittent	5 (17%)	32 (65%)				
Feeding type	Open	21 (72%)	43 (88%)	0.095	2.73	0.839	8.886
	Closed	8 (28%)	6 (12%)				

In contrast, feeding system (open vs. closed) showed a non-significant trend: open systems had a higher number of moderate diarrhea cases (43/64), whereas closed systems displayed a more balanced distribution. Statistical tests did not reach significance (*p* ≈ 0.09), and the odds ratio (OR = 2.73; 95% CI: 0.839–8.886) suggested no clear protective effect of one system over the other.

## 4 Discussion

### 4.1 Interpretation of the findings and comparison with existing literature

This study evaluated the clinical impact of continuous versus intermittent enteral feeding (EF) in patients with Clostridioides difficile infection (CDI)-induced diarrhea. Among 78 patients, continuous feeding was used in 53% and intermittent in 47%. Most (82.1%) were managed via open systems, reflecting common practice in resource-limited hospitals. While no severe diarrhea occurred, 37.2% had mild and 62.8% moderate symptoms, highlighting CDI’s ongoing gastrointestinal burden despite treatment. Antibiotic (89.7%) and PPI (83.3%) exposure was high—well-known contributors to CDI onset and recurrence. The mean hospital stay (38.6 ± 51.5 days) underscores CDI’s clinical and economic toll.

Continuous feeding was associated with significant increases in serum sodium (139.9 → 142.0 mmol/L, *p* = 0.003) and stable potassium (3.9 mmol/L, *p* = 0.006), contrasting with the expected hyponatremia and hypokalemia in diarrheal disease. This suggests effective fluid and electrolyte management consistent with the Infectious Diseases Society of America / Society for Healthcare Epidemiology of America (IDSA–SHEA) CDI guidelines (10). The absence of marked electrolyte disturbances likely reflects mild-to-moderate CDI severity and proactive supportive care, paralleling previous reports (35).

Serum phosphate rose non-significantly (*p* = 0.157), consistent with the heterogeneous literature (36). WBC count increased significantly (11.1 → 12.1 × 10^3^/μL, *p* < 0.001), aligning with established evidence linking leukocytosis to CDI severity (37). Albumin declined (27.4 → 26.7 g/L, *p* = 0.006), reinforcing hypoalbuminemia as a severity marker and predictor of poor outcomes (38, 39). Creatinine rose significantly (117.2 → 131.8 μmol/L, p < 0.001), consistent with IDSA/SHEA severity thresholds (40), while BUN decreased (15.3 → 13.7 mmol/L, *p* = 0.002), likely reflecting successful rehydration. Hemoglobin and glucose remained stable, consistent with uncomplicated CDI.

Overall, continuous feeding maintained biochemical stability, particularly in sodium and BUN, while highlighting ongoing inflammation (WBC), nutritional compromise (albumin), and renal strain (creatinine). These results suggest continuous EF, combined with vigilant hydration, is safe and clinically effective in CDI patients, especially within tertiary care settings.

In contrast, intermittent feeding was associated with significant increases in creatinine (116.5 → 140.8 μmol/L, *p* = 0.000) and decreases in sodium (137.8 → 137.0 mmol/L, *p* = 0.030), albumin (29.8 → 28.9 g/L, *p* = 0.001), hemoglobin (10.6 → 9.9 g/dL, *p* = 0.005), and BUN (*p* = 0.020), indicating mild dehydration and nutritional compromise (41, 42). The higher prevalence of BUN/Creatinine > 20 (70.6% vs. 51.9%) in intermittently fed patients—though not statistically significant (*p* = 0.178)—suggests a greater risk of prerenal azotemia, consistent with earlier findings (43, 44).

Diarrhea severity differed markedly by feeding strategy: moderate diarrhea occurred in 65.3% of intermittently fed vs. 34.7% of continuously fed patients (p = 0.000), consistent with evidence linking intermittent feeding to higher gastrointestinal intolerance (45). Continuous feeding increased the odds of mild diarrhea eightfold (OR = 7.91, *p* < 0.001), confirming a protective effect.

Formula category showed no significant association with diarrhea severity (*p* = 0.617), aligning with literature emphasizing fiber composition—rather than brand—as a key determinant (26). The lack of brand-based differences may stem from broad grouping, masking variations in fiber and osmolarity (46). Antibiotic–PPI co-use was prevalent (91.4%) and strongly associated with CDI risk (OR = 74.7, 95% CI 7.82–712.83), corroborating recent evidence of a synergistic effect (47, 48).

In summary, continuous feeding was associated with better fluid-electrolyte stability and lower diarrhea severity compared with intermittent feeding. While both methods are viable, continuous EF appears more protective in CDI, consistent with meta-analytic findings (45, 49, 50). Diarrhea remains multifactorial, influenced by feed type, medications, and infection dynamics, but feeding strategy represents a modifiable component of optimized CDI management.

### 4.2 Limitations

The retrospective design of the study limits the ability to establish causality between enteral feeding strategies and CDI-induced diarrhea. Also, the reliance on medical records for data collection may introduce potential biases, such as missing or incomplete data. Moreover, the study does not assess the impact of specific enteral feeding formulas on CDI-induced diarrhea, which could be potential areas for future research.

### 4.3 Implications for clinical practice and research

This study indicates that enteral feeding delivery methods can significantly influence the development and course of nosocomial *Clostridioides difficile*–associated diarrhea. Clinically, selecting feeding strategies tailored to individual patient risks may reduce gastrointestinal complications, improve nutritional tolerance, and prevent issues such as electrolyte imbalances or aspiration. Regular monitoring of laboratory and nutritional markers can enable early identification of at-risk patients and timely adjustments in feeding, supporting recovery and reducing hospital burden. From a research perspective, these results underscore the need for larger, prospective, randomized studies to confirm these findings, clarify the biological mechanisms linking feeding strategies to CDI, and guide evidence-based nutritional care in hospitalized patients.

## 5 Conclusion

This study highlights the impact of enteral feeding strategies on CDI-induced diarrhea in hospitalized patients, demonstrating that continuous feeding is associated with significantly reduced diarrhea severity compared with intermittent feeding. Patients receiving continuous feeding were nearly eight times more likely to experience mild rather than moderate diarrhea, indicating better gastrointestinal tolerance. Continuous feeding also supported biochemical stability, particularly sodium balance, whereas intermittent feeding was linked to increased creatinine and declines in albumin, hemoglobin, and sodium, reflecting greater renal and nutritional stress. Prognostic markers such as leukocytosis, hypoalbuminemia, and elevated creatinine were reinforced. Concurrent antibiotic and PPI use was highly prevalent, underscoring the need for improved prescribing stewardship. Overall, continuous enteral feeding appears clinically advantageous in CDI management, and these region-specific findings support further prospective studies to refine nutritional strategies and multidisciplinary care approaches.

## 6 Recommendations and future directions

In managing CDI patients on enteral nutrition, continuous feeding should be prioritized as it may reduce diarrhea severity and support biochemical stability, while intermittent feeding requires caution, particularly in nutritionally compromised or hydration-sensitive individuals. Routine biochemical monitoring—including albumin, hemoglobin, BUN, creatinine, and electrolytes—is essential to detect early signs of dehydration, nutritional decline, or renal stress. Antibiotic and PPI stewardship is critical, with careful evaluation of PPI necessity alongside antibiotics to limit CDI risk, coupled with deprescribing when not clinically indicated. Diarrhea management should be comprehensive and multidisciplinary, ensuring that other causes such as medications or infections are excluded before altering feeding regimens. Although the current findings showed no significant association, closed feeding systems remain preferable for their lower contamination risk, provided strict hygiene is maintained when open systems are used.

Future research should explore long-term outcomes, formula composition, and tube system variables through well-designed prospective studies, including multicenter trials, to clarify causal relationships and inform evidence-based feeding protocols that minimize CDI risk without compromising patient care.

## Data Availability

The data analyzed in this study is subject to the following licenses/restrictions: The datasets analyzed during the current study are available from the author on reasonable request. Requests to access these datasets should be directed to rykhattab@iau.edu.sa.
